# Is internet-based cognitive behavioral therapy for alcohol use disorder equally effective for men and women? Implications of a secondary analysis of a clinical trial

**DOI:** 10.3389/fpsyt.2024.1486278

**Published:** 2024-12-20

**Authors:** Greta Schettini, Magnus Johansson, Sam Andersson, Danilo Romero, Anne H. Berman, Philip Lindner

**Affiliations:** ^1^ Centre for Psychiatry Research, Department of Clinical Neuroscience, Karolinska Institutet, Stockholm, Sweden; ^2^ Stockholm Centre for Dependency Disorders, Stockholm Health Care Services, Region Stockholm, Stockholm, Sweden; ^3^ Department of Psychology, Uppsala University, Uppsala, Sweden

**Keywords:** addiction, gender-difference, sex-difference, alcohol, digital interaction, cofounders

## Abstract

**Introduction:**

Excessive alcohol use is a major public health concern, for which internet interventions have shown to be effective. Group-average effects may however mask substantial inter-individual variations in changes; identifying predictors of this variation remains an important research question. Biological sex is associated with pharmacokinetic differences in alcohol tolerance, which is reflected in many national guidelines recommending sex-specific thresholds for excessive drinking. Whether effects of internet interventions are moderated by sex, and whether any moderation is due to confounders, remains largely unexplored.

**Aim:**

To examine sex-differences in outcomes (both response and remission) after an internet intervention for alcohol use disorder, and to identify any confounders.

**Method:**

The current study is a secondary analysis of a randomized controlled trial. After identifying factors in which men and women differed at baseline, mixed effects models were re-run using a subsampling matching strategy.

**Results:**

Men and women differed in baseline sum of drinks and self-rated anxiety. Sex was found to moderate (absolute) response but not remission, neither when using sex-specific or common thresholds for risky drinking. However, after controlling for baseline drinking through subsampling, the difference in response was no longer significant.

**Conclusion:**

Our findings suggest that the apparent sex-difference in treatment response was confounded by intercept-slope correlation – i.e. since men on average drank more at baseline, this offered larger room for decreasing. When conducting studies on internet interventions for addictive disorders, it is crucial to consider which outcomes to use, and how these are operationalized.

## Introduction

Globally, alcohol is one of the greatest risk factors for deaths and causes substantial health loss among the world’s population (1, 2). Even though there are several efficient ways to prevent and treat problematic alcohol use (3–5), only approximately 15% of individuals with alcohol use disorder seek and receive help (6). This constitutes one of the largest treatment gaps among mental disorders (7). Internet-based interventions for problematic alcohol use have emerged as an alternative to traditional face to face treatments (8–10). Availability and anonymity appear to make this option attractive to sufferers (11–14), creating a potential to attract those who would not otherwise seek help (15–17). Meta-analyses have revealed these interventions to be efficacious (8, 18), even comparable in effects to traditional face-to-face treatments (19).

However, as in traditional alcohol interventions (3–5), significant group-average decreases in drinking and symptom scores can mask substantial inter-individual variations in change (17). Past research has shown that the population of individuals with alcohol problems shows substantial heterogeneity with regards to many key characteristics (1, 20), which could reasonably be expected to moderate outcomes of an internet intervention. Indeed, matching individuals to different alcohol interventions has been a topic of some past research, dating back over twenty years (21–23).

One dimension well-known to moderate the presentation of many psychiatric disorders (24, 25), preferences for treatment (26) and even treatment outcomes (27), is sex. We recognize that it is often unclear whether studies within these field use sex (what sex one is assigned to at birth), or gender (which can be the same as what one is assigned to at birth, but also differ from it). Since this distinction is not made in most of the extant literature, we have opted to use sex consistently where the distinction is not clearly stated, since sex-specific guidelines on drinking are grounded in biological, pharmacokinetic sex-difference (28), but also recognize that this is a simplification. There is robust evidence that men, on average, drink more than women, and are over-represented in addiction care (1, 20), but whether sex is also a moderator of the effects of treatment intervention has received little attention in the extant literature. There are some studies suggesting that women with problematic alcohol use benefit from interventions that encompass recognition of gender expectations and the stigma of not living up to the results (29). A meta-analysis that investigated moderators of outcomes of internet-based alcohol interventions (30), concluded that data on sex were limited, particularly women, but that five studies that did investigate this failed to find that gender modified the difference in alcohol consumption between the trial arms. A later, individual-patient meta-analysis instead found that gender was a moderating factor, where females decreased their mean weekly standard units less than men (18). These results did not remain significant when imputing missing values, yet to what degree the imputation technique took gender into account, was not reported. 

An often-overlooked dimension when examining sex-moderated outcomes of alcohol interventions, is that the outcome measure may *in-itself* be sex-moderated. As per clinical trial methodology, one needs to distinguish between *change* and *final state*. The former is captured by continuous measures such as *reduction* and *response* (typically denoting numeric decreases in symptom ratings, either absolute or relative, respectively), while the latter is captured by ratios of participants above or below a prespecified threshold, as in the case of *remission*. Importantly, the fact that men on average drink more than women, has important, but often neglected consequences for both types of outcomes. Many studies (31–33), including our own, have for example relied on national guidelines to threshold drinking into risk- and non-risky. In many countries, these guidelines are sex-specific (34), with the previous Swedish guidelines for example allowing men to drink 55% more standard units per week than women. Whether the average sex-difference in baseline drinking between men and women is equal, either in absolute or relative magnitude, to the sex-difference in remission thresholds, is not typically reported. Even if so, this assumes equidistance of change scores, i.e. that a 7-drink reduction from 22 drinks to 15, is the same as from 15 to 8. Importantly, it is mathematically impossible to match equidistance in both relative and absolute terms at the same time, assuming there is a baseline difference. This means that a baseline sex-difference may also confound numeric outcome measure such as reduction and response (henceforth used synonymously).

A baseline sex-difference in drinking may thus be a confounder in examining moderating effects of sex on treatment outcomes. Third-variable confounding (e.g. in psychiatric comorbidity) complicate the issue further. This highlights a potential concern: if studies investigate whether a treatment’s effectiveness differs by gender but rely solely on the number of drinks consumed as an outcome measure, they may mistakenly interpret a difference as treatment-related. However, this apparent difference might actually stem from baseline values or cofounders rather than the treatment itself. Therefore, the hypothesis of this study is that there will be a significant difference between genders, but this difference may be explained by baseline variations and/or other confounding factors.

In sum, there are inconsistent findings in the extant literature as to whether internet-based interventions for alcohol use disorder have different effects for men and women and to our knowledge, no previous study examining sex-moderation of outcomes in interventions for a problematic alcohol use has systematically examined confounding. It remains unknown whether previous positive findings were due to confounding. To examine this important question, we performed secondary analyses of a randomized controlled trial.

## Methods

### Ethics

The RCT from which data was used, was approved by the Swedish Ethical Review Authority (no. 2014/1758-31/2) and all participants provided digital informed consent. Additional, secondary analyses for the purpose of the current study were also approved by the Swedish Ethical Review Authority (2022-01019-02).

### Data

This study is a secondary analysis of data from a three-arm randomized controlled trial (35) which investigated the effects of a web-based alcohol program with or without therapist guidance among anonymous adult help-seekers. The participants (n=1169) were individuals with a harmful use of alcohol [defined as >15 total score in AUDIT (36), the gold standard screening test for problematic alcohol use, with good psychometric properties (37)] or alcohol dependence (defined as 3 or more ICD-10 criteria). The participants were randomly assigned to an internet-delivered CBT program as self-help (i.e. texts and videos based on motivational interviewing (38), relapse prevention (39), and behavioral self-control (40) followed by checklists and open questions), an internet-delivered CBT program with therapist guidance (the same program as the self-help iCBT group, with a therapist giving feedback on what the participants wrote and registered), or information control in a ratio of 1:1:1. Baseline data, including birth sex and gender, drinking pattern, depression, anxiety, and quality of life, were collected before the participants were randomly assigned (the full demographic variables are shown in **Appendix 1**). Follow-ups were conducted 3 and 6 months after allocation, with the primary outcome being self-reported standard drinks per week, with AUDIT scores serving as secondary outcome. The results showed that the therapist-guided program significantly reduced both weekly drinking and AUDIT scores more than the information control, that the self-help program significantly reduced AUDIT scores more than the information control but not weekly drinking, and that there were no significant differences in either weekly drinking or AUDIT score between the therapist-guided and self-help programs. The attrition was 49% at 3-month follow-up. For more details on participant recruitment, procedure, interventions and full outcomes, see the primary trial reporting (35).

At baseline, participants provided data on both their assigned sex at birth (man or woman), and their gender identity (several options). Concordance rate was calculated to 97.4%. Since national drinking guidelines are exclusively based on biological sex, in turn grounded in pharmacokinetic differences (28), the assigned sex at birth was used for the moderation analyses herein described.

### Measures

In the current study, the primary measure used was weekly self-reported alcohol consumption, using the timeline follow-back (TLFB) method (41) with the Swedish definition of standard drinks (where one standard drink contains 12 grams pure alcohol). The TLFB data was used to calculate both (absolute) response (continuous), as well as remission, defined as low-risk drinking (categorical). Here, we used both the previous, sex-moderated Swedish guidelines (<10 for women and <15 for men), as well as the current, common Swedish guidelines (<10 for both men and women). In examining potential confounders, we examined both raw scores of the 10-item AUDIT (36) as well an adapted version omitting the three consumption items. The number of self-endorsed ICD-10 criteria for alcohol dependence (42) was also analyzed, as was self-rated anxiety using the GAD7 (43), depression measured using the MADRS-S (44), and health related quality of life, measured using the EQ5D (45).

### Statistical analyses

Since our goal was to examine whether men and women had different outcomes, the current study only includes the two arms that received treatment (n=777); these arms were collapsed into one since the primary outcome study revealed no difference in outcomes between the two. Importantly, preliminary analyses revealed no three-way interactions between time, gender, and whether therapist-support was provided or not, when the two treatment arms were directly contrasted. Moreover, since the primary outcome study found that the treatment effect was observable at the three-month assessment, only two timepoints (pre- and post-treatment) were included in the secondary analyses to simplify modeling and interpretation of parameters.

First, we used t-tests to examine which potential baseline confounders (including baseline drinking) were associated both with sex and decrease in drinking after treatment. Using a subsampling matching strategy that involved dropping either the top or bottom 10% from each sex for each respective confounder, we then re-ran our random-intercept, time × sex linear mixed effect model, using the matched subsample and compared findings. For linear mixed effects models, bootstrapped confidence intervals were calculated to account for non-normal distribution of residuals due to excess zeros post-treatment.

Next, since the association between baseline drinking and subsequent decrease in drinking was of *a priori* interest, we performed quantile regression (46), with the former as predictor and the latter as outcome, with quantiles 0.2-0.8 in steps of 0.2, and compared intercept and beta estimate quantile curves across sex. This was first done using the whole sample with sex as an additional predictor, including the interaction term. Next, analyses were repeated for each sex separately. These supplementary analyses were performed on complete data only (n=383), as not to risk neither introducing nor neglecting sex-specific associations in any imputation.

## Results

### Potential baseline confounders

Analyses revealed that at baseline, men and women differed significantly in mean weekly drinks and mean GAD-7 scores (see **Table 1**). No significant differences in mean MADRS scores, EQ5-D scores, self-endorsed dependency symptoms, or AUDIT scores (either raw, or consumption items omitted), were found.

**Table 1:** Baseline descriptives for women and men.

	Women (n=448)		Men (n=329)		T statistics (df=777)	
Measure	M	SD	M	SD	t	p
AUDIT	21.96	5.87	22.43	5.17	1.158	0.247
AUDIT item 4–10*	13.84	4.82	13.90	4.30	0.196	0.844
Dependence	4.23	1.31	4.31	1.36	0.861	0.389
EQ5index	1.53	11.06	1.10	5.30	-0.644	0.520
GAD7	8.97	5.50	7.77	5.15	-3.083	0.002
MADRS	18.76	8.95	18.04	9.24	-1.104	0.270
Weekly drinks	22.67	14.25	29.09	19.59	5.292	<.001

*AUDIT item 4–10 refers to the adapted AUDIT score without the three consumption items.

### Remission outcomes

Logistic mixed effects modeling revealed no significant time × sex effects on remission outcomes, either when using sex-specific thresholds for low-risk drinking (95% CI: -0.65—0.88) or the common threshold (95% CI: -0.37—0.944). Hence, there was no effect for which to consider confounding.

### Response outcome

In the raw mixed effect model, there was a significant time × sex effect such that men decreased their drinking more than women (B=5.85, 95% CI: 2.35—9.62), departing from a greater baseline level (B=6.42, 95% CI:-8.61—4.32). Posthoc testing using estimated marginal means revealed no between-group difference at post (p=0.727). When re-running this analysis using the matched subsample, neither the baseline difference in drinking (B=0.92, 95% CI: -1.00—2.99) nor the time × sex effect (B=-0.49, 95% CI: -3.75—2.58) remained significant, suggesting that the apparent sex-difference in decreased drinking was not driven by sex per se, but by an omnibus slope-intercept correlation.

**Figure 1:**
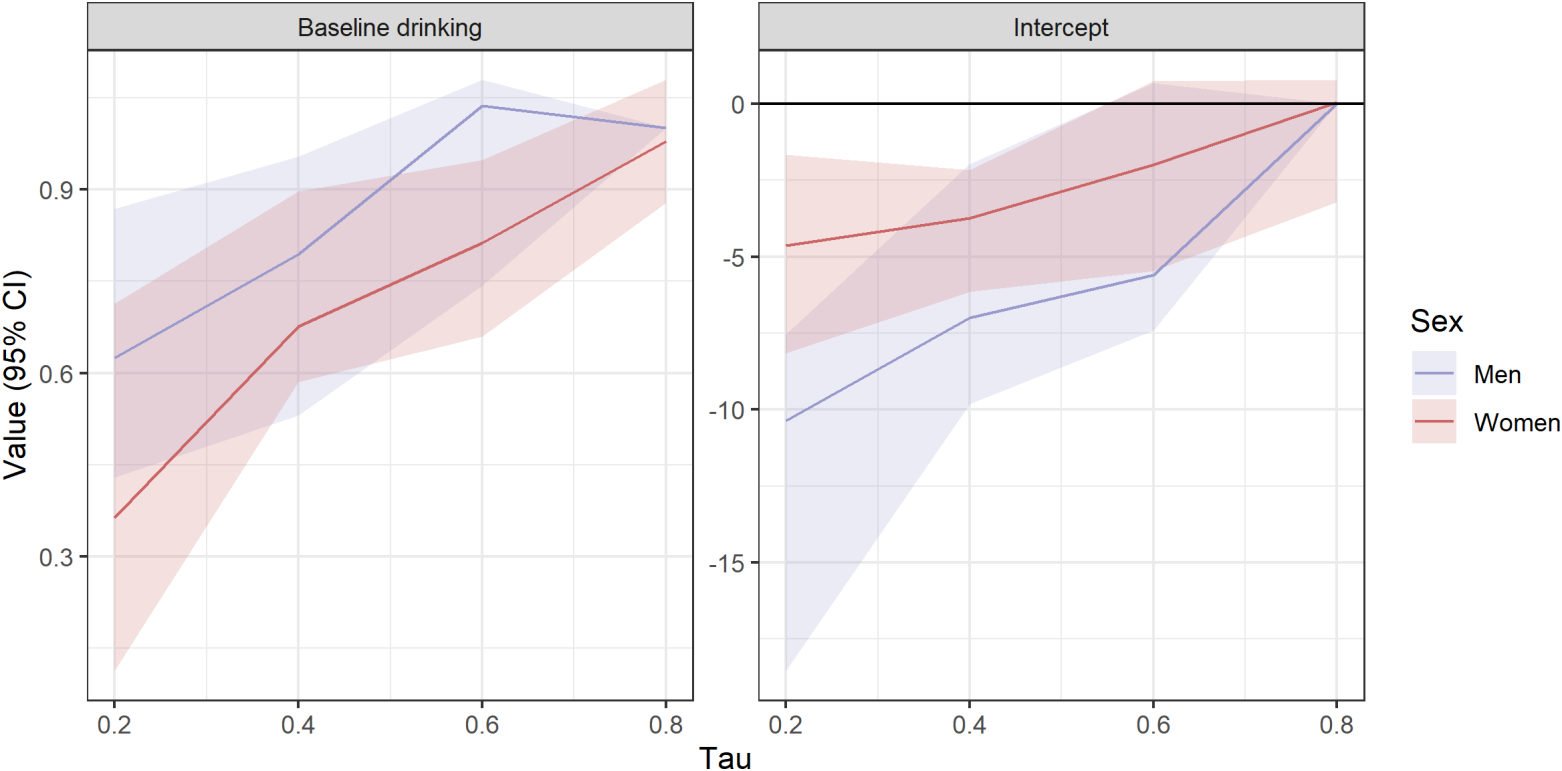
Quantile regression plots by sex. One upper bound value replaced with lower bound equivalence due to convergence error. Note that the dependent variable (decrease in drinking) was calculated by T0 scores minus T1 scores, entailing that a true decrease corresponds to a positive value.

Further analyses with quantile regression using the full sample revealed a significant baseline drinking × sex effect on decreased drinking only on the 0.8 quantile (B=-0.021, 95% CI: -0.089—0.011), but this was likely due to a convergence error. Congruently, examining sex-specific intercept and estimate curves across quantiles. See **Figure 1**.

In examining the possible confounding effect of baseline anxiety on sex-differences in decreased drinking, we transformed the (numeric) baseline GAD-7 scores into a binary (time-invariant) predictor of high baseline anxiety using a median-split approach; this was done in order to avoid assuming linear two-way interaction effects. Although subsequent mixed effects modeling did reveal that there was indeed a significant time × anxiety effect (95% CI: 4.28–15.13), those with high baseline anxiety also had higher baseline drinking (95% CI: 2.83–9.45) and there was no significant time × anxiety × sex effect (95% CI: -11.05–3.25) that would have revealed differential treatment effects between the sexes in cases of comorbid high anxiety.

## Discussion

The current study replicated past research in showing differential treatment outcomes between men and women when examining response (32), but not remission (33). However, after taking a baseline difference in drinking into account through a subsampling strategy, the difference in response was no longer significant, suggesting that this apparent sex-difference was confounded by intercept-slope correlation – i.e. since men on average drank more at baseline, this offered larger room for decreases.

Our findings stress the importance of carefully considering which outcome that best captures the desired change after treatment, as well as how other study characteristics impact outcome modeling. First, the definitions of both low-risk drinking and hazardous drinking differ greatly between countries – and in most countries, the definition of low-risk drinking also differs by sex (34). A direct consequence of this sex-based target differentiation is that women must reduce their drinking more to achieve low-risk drinking, assuming they start from the same baseline level. This, in turn, is however seldom the case (including in the current study). In several studies similar to ours, sex-specific definitions are also used as outcome, with inconsistent results. For instance, some studies on predictors of change in internet interventions have not found any significant difference among women (47) or somewhat better results among women (48). But in a study that investigated predictors of change in a similar intervention as the current study, women were found to be less likely to have low-risk consumption at follow-up compared to men when previous Swedish sex-specific guidelines were used as outcome (32). These results were replicated a few years later (49). A more recent study, exploring the effects of a web-based intervention for alcohol and PTSD symptoms among veterans, also showed that significantly fewer women achieved low-risk drinking after one-month, but also that women did not reduce their weekly drinking as much as men after six months (33). These results on continuous drinking outcome are similar to the findings in our study and the findings from the previously mentioned individual data meta-analysis by Riper et al. (18). In a British study investigating the predictors of outcomes of a mobile app targeting harmful alcohol use, the only predictor associated with the extent of alcohol reduction was how much the participant drank at baseline (50), similar to the findings in the current study.

Multiple studies have revealed that both the sensitivity and specificity of the sex-specific definitions have had large variation (51, 52). Also, there are ethical aspects in using assigned sex rather than individuals identified gender. Gender is not necessarily binary, and using uniform measures could result in more inclusive standards (53). Further, there has also been an ongoing discussion about using categorical outcomes for alcohol interventions, such as cut-off scores for heavy, or hazardous drinking (54). Unless the explicit target of an intervention is to decrease drinking to a specific, sex-indifferent level [e.g. before planned surgery (55)], capturing change after treatment with an absolute or relative response metric will circumvent this issue; should sex-differences in outcomes be of special interest, analyses should then preferably be adjusted similar as to in the current study. Of note, this applies only when considering any change in drinking as clinically meaningful: if total abstinence (i.e. a naturally occurring zero) is the only intended outcome, the entire issue of sex-specific outcomes is largely rendered irrelevant.

Strengths of the current study are that the sample is both large and inclusive. Another strength is that a multitude of confounders were considered. There are also several limitations to the study. First, we opted to focus on total number of standard units per week, since this is the most common outcome in the field of digital interventions for alcohol problems, and also the main metric (along with daily drinks) on which national drinking guidelines are based. Similar analyses could also be performed for other TLFB-derived metrics like drinking days, average number of drinks per drinking day, days with binge drinking, maximum drinks on any given day, and other clinically pertinent metrics. Second, it was deemed out of scope in the current study to examine whether popular imputation techniques for missing data should be performed separately by sex. Third, the current study did not attempt to associate change in drinking to treatment adherence; such analyses would however need to account for the non-randomized nature of this variable, which has shown to be associated with baseline severity in at least one other study on internet interventions for addictive disorders (56).

Considering the magnitude of the alcohol problem, and that iCBT already has a proven track record of reaching and attracting large samples, there are excellent reasons to continue developing and evaluating the effects such similar interventions not only on a group-level, but also subgroup-level. In choosing which potential moderators to examine, it is important that these are anchored in evidence and proper deductions that show why these may indeed moderate outcomes, as to avoid Type 1 errors through involuntary *hypothesizing after the results are known* (57). Findings of the current study highlight the importance of carefully consider which outcomes to specify when conducting studies on internet interventions for addictive disorders which accept both sexes.

## Data Availability

The raw data supporting the conclusions of this article will be made available by the authors, without undue reservation.

## Appendix 1

**Table:** Demographics of the participants.

		Women (n=448, 57.66%)	Men (n=329, 42.34%)	Total (n=777)
Education				
	University or college	254 (56.7%0)	152 (46.20%)	406 (52.25%)
	Upper secondary school, high school or equivalent	159(35.49%)	147 (44.68%)	306 (39.38%)
	Primary school or folk school	25 (5.58%)	27 (0.20%)	52 (6.69%)
	Other	10 (2.23%)	3 (0.91%)	13 (1.67%)
Residence				
	Villa or townhouse	180 (40.18%)	140 (42.55%)	320 (41.18%)
	Rental apartment/room	159 (35.49%)	109 (33.13%)	268 (34.49%)
	Condominium	101 (22.54%)	76 (23.10%)	177 (22.78%)
	Other	8 (1.79%)	4 (1.22%)	12 (1.54%)
Living circumstances				
	With partner and child(ren)	156 (34.82%)	121 (36.78%)	277 (35.65%)
	With partner only	110 (24.55%)	99 (30.09%)	209 (26.90%)
	With child(ren) only	44 (9.82%)	11 (3.34%)	55 (7.08%)
	Alone	75 (16.74%)	63 (19.15%)	138 (17.76%)
	Other	63 (14.06%)	35 (10.64%)	98 (12.61%)
Civil status				
	Married	158 (35.27%)	127 (38.60%)	285 (36.70%)
	Cohabiting	111 (24.78%)	84 (23.53%)	195 (25.10%)
	Single	111 (24.78%)	87 (26.44%)	198 (25.48%)
	Separated/divorced	64 (14.29%)	30 (9.12%)	94 (12.10%)
	Widow/widower	4 (0.89%)	1 (0.30%)	5 (0.64%)
Source of income				
	Employment	347 (77.46%)	265 (80.55%)	612 (78.76%)
	Study allowance	29 (6.47%)	12 (3.65%%)	41 (5.28%)
	Pension	15 (3.35%)	22 (6.69%)	37 (4.76%)
	Other	57 (12.72%)	30 (9.12%)	87 (11.20%)
Country of birth				
	Sweden	413 (92.19%)	310 (94.22%)	723 (93.05%)
	Other Nordic country	18 (4.02%)	7 (2.13%)	25 (3.22%)
	Rest of Europe	11 (2.46%)	6 (1.82%)	17 (2.19%)
	Outside Europe	6 (1.34%)	6 (1.82%)	12 (1.54%)
